# Comparative Analysis of Pathogenicity and Phylogenetic Relationship in *Magnaporthe grisea* Species Complex

**DOI:** 10.1371/journal.pone.0057196

**Published:** 2013-02-26

**Authors:** Jaehyuk Choi, Sook-Young Park, Byung-Ryun Kim, Jae-Hwan Roh, In-Seok Oh, Seong-Sook Han, Yong-Hwan Lee

**Affiliations:** 1 Center for Fungal Pathogenesis, Seoul National University, Seoul, Korea; 2 Crop Environment Div., National Institute of Crop Science, Rural Development Administration, Suwon, Korea; 3 Department of Agricultural Biotechnology, Center for Fungal Genetic Resources, Plant Genomics and Breeding Institute, and Research Institute for Agriculture and Life Sciences, Seoul National University, Seoul, Korea; Soonchunhyang University, Republic of Korea

## Abstract

Outbreaks of rice blast have been a threat to the global production of rice. Members of the *Magnaporthe grisea* species complex cause blast disease on a wide range of gramineous hosts, including cultivated rice and other grass species. Recently, based on phylogenetic analyses and mating tests, isolates from crabgrass were separated from the species complex and named *M. grisea*. Then other isolates from grasses including rice were named as *M. oryzae*. Here, we collected 103 isolates from 11 different species of grasses in Korea and analyzed their phylogenetic relationships and pathogenicity. Phylogenetic analyses of multilocus sequences and DNA fingerprinting revealed that the haplotypes of most isolates were associated with their hosts. However, six isolates had different haplotypes from the expectation, suggesting potential host shift in nature. Results of pathogenicity tests demonstrated that 42 isolates from crabgrass and 19 isolates from rice and other grasses showed cross-infectivity on rice and crabgrass, respectively. Interestingly, we also found that the isolates from rice had a distinct deletion in the calmodulin that can be used as a probe.

## Introduction

Rice (*Oryza sativa*) is arguably the most important staple food crop in the world, contributing ∼30% of nutritional intake of the world’s population [1]. Outbreaks of rice blast disease have been a constant threat to the world cereal production. *Magnaporthe oryzae* is the most prominent cause of blast disease on a broad range of grasses including rice as well as other species of Poaceae [2]. To date, 137 members of Poaceae hosting this fungus have been described in Fungal Databases (http://nt.ars-grin.gov/fungaldatabases/, updated on Apr. 6, 2012) [3]. Since individual isolates have a limited host range [4], they were regarded as the *Magnaporthe grisea* species complex (*Mg* complex).

Two centuries ago, this pathogen was first isolated from crabgrass *(Digitaria sanguinalis*) and was named as *Pyricularia grisea* [5]. Another name, *Pyricularia oryzae* has also beenused since Cavara identified an isolate from rice in 1892 (Cavara, Fungi Longobardiae #49). Although slight morphological differences between them were noted, the differences were not considered sufficient to differentiate them. Consequently, both names were used synonymously for these species without a clear means to distinguish them. Even then, Sprague [6] noted that, despite the difficulty in morphological distinction, rice was predominantly observed to be the host of *P. oryzae* in the literature available at the time. After successful mating of these species was observed [7], [8], *M. grisea* was proposed to use as the name for representing the *Mg* complex according to the rules of nomenclature [9].

In fungi, the morphological species concept (MSC) is the most prevalent method of diagnosing species because morphological traits of individuals are readily detectable and comparable [10]. For example, ascus (meiospore) and ascocarp (structure containing asci) morphology are important diagnostic criteria in the phylum Ascomycota [11]. The genus *Magnaporthe*, which consists of five species (*M. grisea*, *M. oryzae, M. salvinii*, *M. poae* and *M. rhizophila*) has shared morphological traits such as three-septate fusiform ascospores and black nonstromatic perithecia (ascocarp) with long hairy necks [12]. Discontinuities in morphological characters have been used to delimit species. *M. poae* and *M. rhizophila* produce ‘*Phialophora*-like’ conidiopores and infect only roots of hosts while *M. salvinii*, *M. grisea* and *M. oryzae* form ‘*Pyricularia*-like’ (or sympodial) conidiophore and infect stems or leaves of hosts (*M. oryzae* can also infect rice though its roots) [13]–[15]. *M. salvinii* produces sclerotia in the tissues of host plants that release conidia while *M. grisea* and *M. oryzae* do not have any sclerotium [14], [15]. However, no detectable morphological character exists between *M. grisea* and *M. oryzae*.

The biological species concept (BSC) delimits species clearly based on the formation of meiospores. However, BSC cannot be easily applied to fungi that rarely reproduce sexually [10], [16]. Although the teleomorph of the *Mg* complex was found from laboratory matings [7], [8], sexual reproduction has rarely been observed in nature and fertile strains were found in some isolates from the limited hosts (e.g. *Eleusine* spp.) [17]. In addition, ascospores from the crosses within the *Mg* complex usually germinate with low frequency [17]. As shown in the large scale mating tests, ∼50% of the tested isolates produced perithecia (ascocarp) but less than 5% of them contained viable ascospores [18].

DNA fingerprinting has been used to identify genotypes that are associated with various hosts in the *Mg* complex, using repeat sequences such as Magnaporthe grisea repeat (MGR) 586, grasshopper retroelement, and Magnaporthe gypsy element [19]–[21]. For example, fingerprinting with the MGR586 sequences is useful to identify isolates from rice [19]. Isolates from rice exhibit multiple bands after hybridization with MGR586 sequences while those from other grasses have fewer or no bands according to the number of repeat sequences in each genome [22]–[24]. Thus, the DNA fingerprinting technique has been used to characterize genetic variability within the *Mg* complex [17], [25]–[28]. Recently, the phylogenetic species concept (PSC) based on the concordance of multilocus DNA sequence data has become popular among filamentous fungi [10], [29]. In the *Mg* complex, phylogenetic analyses using actin, beta-tubulin, and calmodulin gene sequences resolved isolates from crabgrass as a distinct phylogenetic group from the other isolates from rice and other grasses [30]. The group of crabgrass isolates was called *M. grisea*, following the original specimen. The isolates from other grasses including rice were named as *M. oryzae* by the authors [30]. Zellerhoff *et al*. (2006) found that the isolates from *Pennisetum* spp. formed a lineage closely related to *M. grisea* [31]. More recently, Hirata *et al.* (2007) suggested the existence of five more phylogenetic species (including three morphological species) in the genus *Pyricularia* [32].

The host range of a pathogen is determined by inoculation tests on different plants. Between rice and crabgrass, however, neither constant nor comprehensive results have been found from several pathogenicity tests for cross-infectivity [2]. As shown in Ou’s review, cross-infectivity might exist between rice and crabgrass: Hori [33] and Suzuki and Hashimoto [34] observed that rice isolates could infect crabgrass; Kawakami [35] and Hemmi *et al*. [36] found that crabgrass isolates could infect rice. Recently, two crabgrass isolates (MG102 and NI907) in Korea and Japan exhibited a typical blast symptom on the leaves of rice and Italian ryegrass, respectively [37], [38]. One rice isolate (1836-3) caused blast lesions on crabgrass [23]. In contrast, the reports of Sawada [39], McRae [40], and Nisikado [41] indicated that rice isolates could not infect crabgrass (reviewed by Ou [2]). No cross-infection between rice and crabgrass was found in the reports where three crabgrass isolates and 22 rice isolates were used for pathogenicity tests [38], [42], [43]. It was proposed that these contradictory results might come from different genetic backgrounds of the isolates and hosts or environmental conditions in the tests [2].

The objectives of this study are (i) to characterize phylogenetic diversity within the *Mg* complex, (ii) to determine if cross-infectivity exists between rice and crabgrass, and (iii) to understand the relationship of haplotypes and host origin.

## Materials and Methods

### Fungal Isolates and Culture Conditions

During the period of 1995–2006, 84 isolates of the blast fungus were collected from 33 sites distributed over Korea (Table S1). Seventy were isolated from crabgrass (*Digitaria sanguinalis*) while 14 were from nine different species of Poaceae. All isolates were purified by single spore isolation and deposited in the Center for Fungal Genetic Resources (Seoul National University, Seoul, Korea; http://genebank.snu.ac.kr). In addition, 13 rice isolates collected in Korean fields were obtained from the Center for Fungal Genetic Resources. Six *M. oryzae* mating type standard strains (70-15, 70-6, 4091-5-8, 4136-4-3, 2536, and Guy11) were generously provided by Dr. Valent (Kansas State University). Fungal isolates were cultured on oatmeal agar media (50 g of oatmeal per liter) or V8 juice agar (8% V8 juice) at 25°C under continuous fluorescent light.

### Ethics Statement

No specific permits were required for the described field studies. The location is not privately-owned or protected in any way. The field studies did not involve endangered or protected species.

### Pathogenicity Test

Conidia were harvested from 7-day-old cultures with sterile distilled water and the concentration was adjusted to 1×10^5^ conidia per ml after filtration through two layers of Miracloth™ (CalBiochem, San Diego, CA). The pathogenicity test was performed by spraying conidial suspension onto four-week-old rice (cv. LTH or cv. Nakdongbyeo) and five-week-old crabgrass grown in the greenhouse (the three- to four-leaf stage). Inoculated plants were kept in the dew chamber in the dark at 25°C for 24 hours and moved back to the greenhouse. Lesions were measured from three plants per strain in an assay and the assay was performed three times. A modified disease index for the blast was used to estimate the virulence of isolates. Lesion types reflecting disease severity were measured 10 days after inoculation according to the rating index described by Valent *et al.* [44]. Then isolates exhibiting susceptible lesions (type 2 to 5) were marked as ‘+’ and the other types (0 and 1) as resistant (‘−’). Disease incidence was displayed with numbers of ‘+’ marks in three trials. For example, one ‘+’ symbol indicates that the susceptible lesions were observed once in three trials. The symbol of ‘++’ means that the lesions were observed two times out of three trials, indicating that disease was observed more frequently or constantly than the case of ‘+’. Images of conidiation on lesions were photographed using a binocular microscope (Leica L2; Leica Microsystem, Germany) with SPOT Advanced software (v3.5.2; Diagnostic Instruments Inc, MI, USA).

### DNA Extraction and Southern Blot Hybridization

Fungal isolates were grown in complete medium (6 g of yeast extract, 6 g of casamino acids, and 10 g of sucrose per liter) in the dark for 4 days. Genomic DNA was extracted from freeze-dried mycelia as previously described [45]. DNA fingerprinting analysis using MGR586 sequence as a probe was performed as previously described [46].

### PCR and DNA Sequencing

DNA amplification reactions were performed for the actin, beta-tubulin, and calmodulin genes as previously described [30]. The PCR reactions were performed with 1 unit of nTaq-Tenuto DNA polymerase (Enzynomics™, Daejeon, Korea) using 20 ng of genomic DNA in a 20 µl of reaction volume and purified using ExoSAP-IT® (USB, Cleveland, OH) following the manufacturer’s instruction. The following primers were used for amplification: ACT-512F (5′-ATGTGCAAGGCCGGTTTCGC-3′) and ACT-783R (5′-TACGAGTCCTTCTGGCCCAT-3′) [47], Bt1a (5′-TTCCCCCGTCTCCACTTCTTCATG-3′) and Bt1b (5′-GACGAGATCGTTCATGTTGAACTC-3′) [48], and CAL-228F (5′-GAGTTCAAGGAGGCCTTCTCCC-3′) and CAL-737R (5′CATCTTTCTGGCCATCATGG-3′ [47]. Sequencing was performed in the National Instrumentation Center for Environmental Management at Seoul National University. PCR products were sequenced using BigDye™ Terminator Cycle sequencing kit (Applied Biosystems, Foster City, CA) following the manufacturer's instruction.

### DNA Sequence Alignment and Phylogenetic Analysis

Sequences of three genes (GenBank accession no. KC167361-669) and their combined sequences were aligned using CLUSTAL W [49] in the MegAlign™ program 5.01 (DNASTAR Inc., Madison, WI). Thirty-one sequences used in a previous study [30] were downloaded from GenBank in the NCBI and used as control in our alignments. The incongruence-length difference for this combined data (1,563 characters) was tested using the partition homogeneity test in PAUP^*^ 4.0 beta 10 [50], [51]. The test was performed with 100 replicates using heuristic searches. Maximum parsimony trees were generated by heuristic searches with the simple addition option and a reference taxon of *M. salvinii* (GenBank accession no. AF395975, AF396004, and AF396030), or with random addition and 500 bootstrap replicates. Pairwise distance for the combined data was calculated in MEGA4 program [52] with p-distance model and 500 bootstrap replicates. A total of 1,250 positions were analyzed after all positions containing gaps and missing data were removed from the dataset. The other options were used with default settings.

## Results

### Multilocus Sequence Typing in the *Mg* Complex

**Figure 1:**
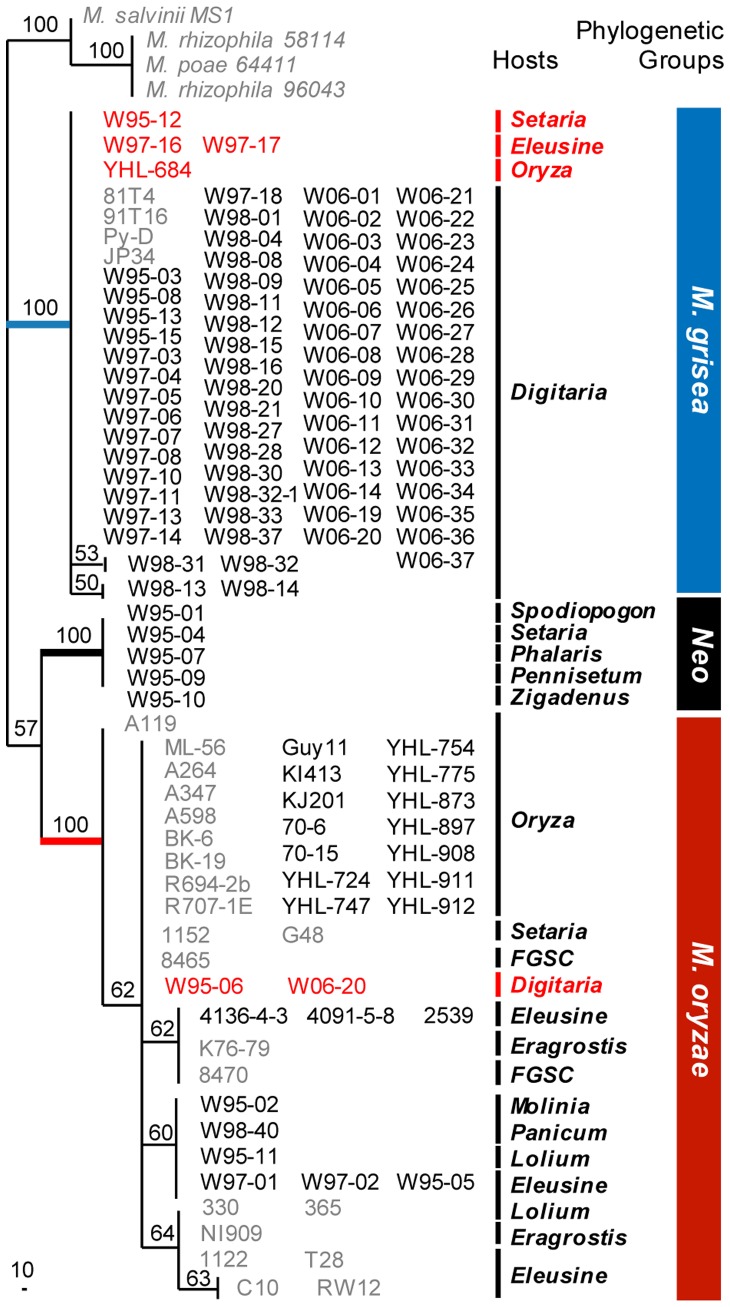
The maximum parsimony tree of *Mg* complex isolates inferred from combined sequences. Labels on the phylogeny are, from left to right: Strain No., host, and the phylogenetic group or species. Sequences used in a previous study [30] were integrated as controls (gray characters). Samples showing inconsistency in haplotype-host origin are indicated in red. The single most parsimonious tree was inferred from 1,563 bp of combined sequence of actin, beta-tubulin, and calmodulin genes including 460 parsimony-informative characters. Bootstrap values, based on 500 replicates, are indicated above the branches. The tree length was 885 steps. The consistency index (CI) and the retention index (RI) were 0.911 and 0.986, respectively.

Multilocus sequence typing (MLST) was performed using actin, beta-tubulin and calmodulin gene sequences. Our collection consists of isolates from crabgrass (N = 70), rice (N = 16), and other grasses (N = 17) (Table 1). In addition, sequence data of 31 isolates used in the study of Couch and Kohn (2002) was included as controls (gray characters in Fig. 1). A single most parsimonious tree (MPT) was generated from the combined sequences of the three genes or three individual genes (Figs. 1 and S1). The tree resolved three clades with high bootstrap values (Fig. 1). Also phylogenetic trees based on individual genes resolved three clades (Fig. S1). In the MPT (Fig. 1), two of the three phylogenetic species included *M. oryzae* and *M. grisea* [30]. The last group consisting of five isolates from other grasses will hereafter be designated as the ‘*Neo*’ group in this study. The relationships of the three phylogenetic groups were incongruent in the MPTs inferred from the individual genes (Fig. S1). In the actin tree, for example, the *Neo* group was outside *M. grisea* and *M. oryzae* while the *Neo* group in the calmodulin gene tree was close to the *M. grisea* (Fig. S1). The tree based on the beta-tubulin sequence showed the same topology with the tree generated using the combined sequences (Fig. 1).

**Table 1:** Hosts of isolates used in this study and their haplotypes inferred from multilocus genealogy.

		Haplotypes based on three genes			
Hosts	No.Isolates	G type a	O type	O2 type	N type
Crabgrass	70	68	0	2	0
Rice	16	1	0	15	0
Other grasses	17	3	9	0	5
Total	103	72	9	17	5

a G: *M. grisea*, O: *M. oryzae,* O2: *M. oryzae* from rice (4 nt missing compared to O type), N: *Neo* group.

To understand the relationship of the three groups, we estimated the genetic distance of these groups using a pairwise analysis of the aligned sequences (Table 2). Representative sequences from the three groups were used for this analysis. Sequences of the isolate from *Pennisetum* sp. (CD 180; GenBank accession no. DQ240880, DQ240912, and DQ240896) were included as a negative control, which formed a lineage closely related to *M. grisea* group in the phylogenetic analysis [31]. The distance of strain CD180 sequence from *M. grisea* was smaller (0.034) than the others (0.088 to the *Neo* group and 0.102 to *M. oryzae*), indicating that strain CD180 was close to *M. grisea* (Table 2). However, the distances between the *Neo* group and *M. oryzae, M. oryzae* and *M. grisea*, and *M. grisea* and the *Neo* group were 0.076, 0.092, and 0.082, respectively (Table 2). Thus, based on its genealogical exclusivity, we concluded that the *Neo* group is a phylogenetic species as independent as *M. oryzae* and *M. grisea*.

**Table 2:** Estimates of evolutionary divergence among three phylogenetic species.

Phylogenetic Species	*M. grisea*	*M. oryzae*	*Neo* group	Control^a^
*M. grisea*	−	(0.008)^b^	(0.008)	(0.005)
*M. oryzae*	0.092	−	(0.008)	(0.008)
*Neo* group	0.082	0.076	−	(0.008)
Control	0.034	0.102	0.088	−

a Strain CD 180 from *Pennisetum* sp. is closely related to *M. grisea* in phylogenetic analysis [31].

b The numbers in parentheses are standard errors.

### A New Method to Identify Rice Origin in the *Mg* Complex

**Figure 2:**
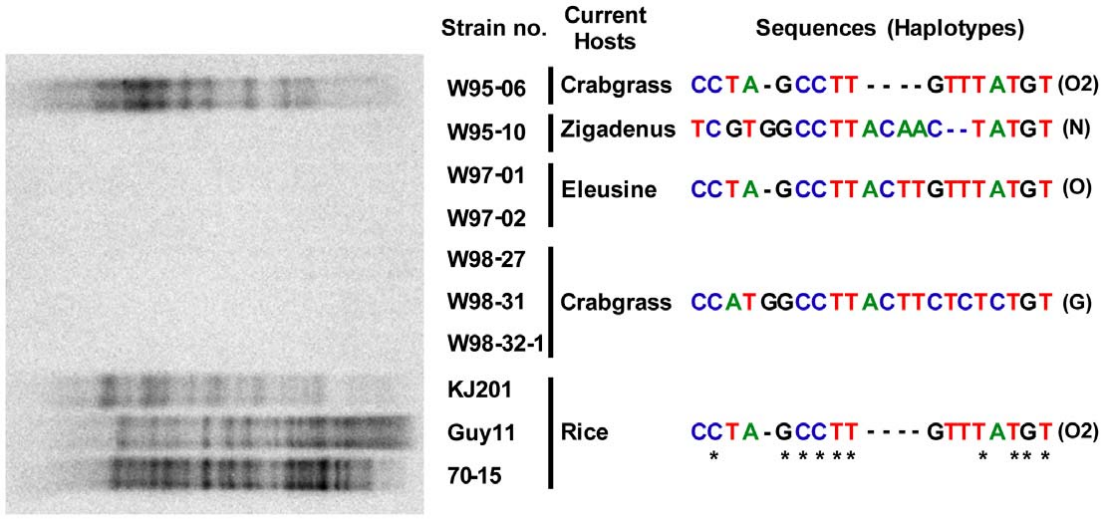
Genotypes of *Mg* complex based on hybridization patterns and calmodulin gene sequences. Repetitive sequence (MGR586) was used as a hybridization probe in Southern blotting (left). Multiple hybridizing bands indicate that the isolate originated from rice [19]. ‘Current hosts’ mean hosts that the isolates were collected from. Nucleotide sequences of the calmodulin gene that differentiate all four haplotypes (right). Asterisks indicate conserved nucleotides among the four haplotypes.

Sequence analysis revealed that a new deletion polymorphism exists within *M. oryzae* (species complex). The calmodulin gene sequence of the rice isolates was 4 bp shorter (469 bp; Genebank ID: AF396024) than that of the known *M. oryzae* type sequence (473 bp; AF396025). Four nucleotides (ACTT) at the 10–13^th^ position of the calmodulin gene were deleted in the sequences of the rice isolates, compared to the other grasses isolates (Fig. 2). No difference was found in the actin and beta-tubulin gene sequences between those isolates. We confirmed that this difference exists in all the sequences of rice isolates publicly available; 11 sequences from Couch and Kohn’s work (2002), 5 from Zellerhoff *et al.*’s work (2006), and 28 from Hirata *et al.*’s work (2007). Thus, these isolates were annotated as ‘O2’ type (Tables 1 and S1). The correlation in nucleotide loss and host origin was further supported by Southern blot analysis with the MGR586 sequence. Twelve out of 18 isolates identified as the ‘O2’ type in Table 1 were tested and they all showed the typical fingerprint of the rice pathogen [19]. Thus, this short polymorphic region in the calmodulin gene can be used to identify rice pathogens as an alternative to the conventional fingerprinting method.

### The Relationship between Haplotype-host Origin

We investigated the relationship between the haplotypes of the three phylogenetic groups and the hosts which they had been isolated from. In crabgrass isolates, 68 out of 70 had the *M. grisea* haplotype (‘G’ type in Table 1). However, two isolates (W95-06 and W06-20) were discovered as having the *M. oryzae* type sequence, specifically with the ‘O2’ type sequence (Table 1). Southern blot analysis using MGR586 as a hybridization probe (Fig. 2) also confirmed that W95-06 had originated from rice because it exhibited multiple bands typical of the rice isolates (KJ201, Guy11, and 70-15). In contrast, three crabgrass isolates (W98-27, W98-31, and W98-32-1) showed no band after hybridization to the probes, indicating that they had not originated from rice (Fig. 2).

**Figure 3:**
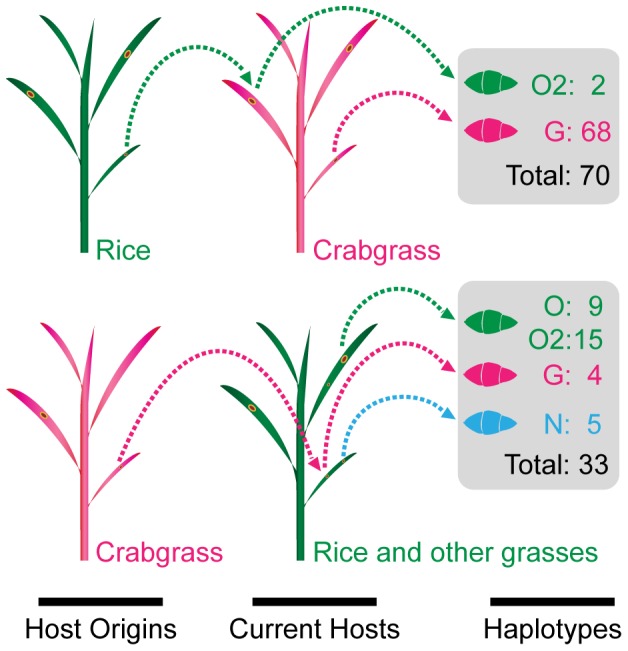
A schematic model for relationship of haplotype-host origin. Cross-infectivity or host shift may cause inconsistency in relationship of haplotype-host origin in the *Mg* complex. Different haplotypes of the isolates were labeled with different colors in their names, movements, spores, and host plants. Haplotype abbreviation: G–*M. grisea*, N-*Neo* group, O–*M. oryzae,* and O2-*M. oryzae* from rice.

Among 33 isolates from rice and other grasses, four had the *M. grisea* haplotype sequence (‘G’ type in Table 1). Remarkably, one of them was strain YHL-684 obtained during a collection of 174 rice field isolates in Korea [46]. In hybridization analysis with MGR586 sequences, strain YHL-684 did not show any band, indicating that it did not originate from rice (data not shown). Contrary to this, the other YHL strains (N = 10) displayed multiple bands in the hybridization analysis (data not shown). Three other isolates with the *M. grisea* type sequence were from *Setaria viridis* (W95-12) and *Eleusine indica* (W97-16 and W97-17). Thus, 24 out of 28 isolates had the *M. oryzae* haplotype as expected according to the isolated hosts, after exclusion of five isolates belong to the *Neo* group (Table 1). In summary, six isolates had haplotypes that were inconsistent with the hosts they were isolated from. Based on this inconsistency in haplotype-host origin relationship, we hypothesized that cross-infectivity or host shift had occurred (e.g. between crabgrass and rice) in the field situation, involving the six isolates (Fig. 3).

### Comparative Pathogenicity Analysis on Rice and Crabgrass

**Figure 4:**
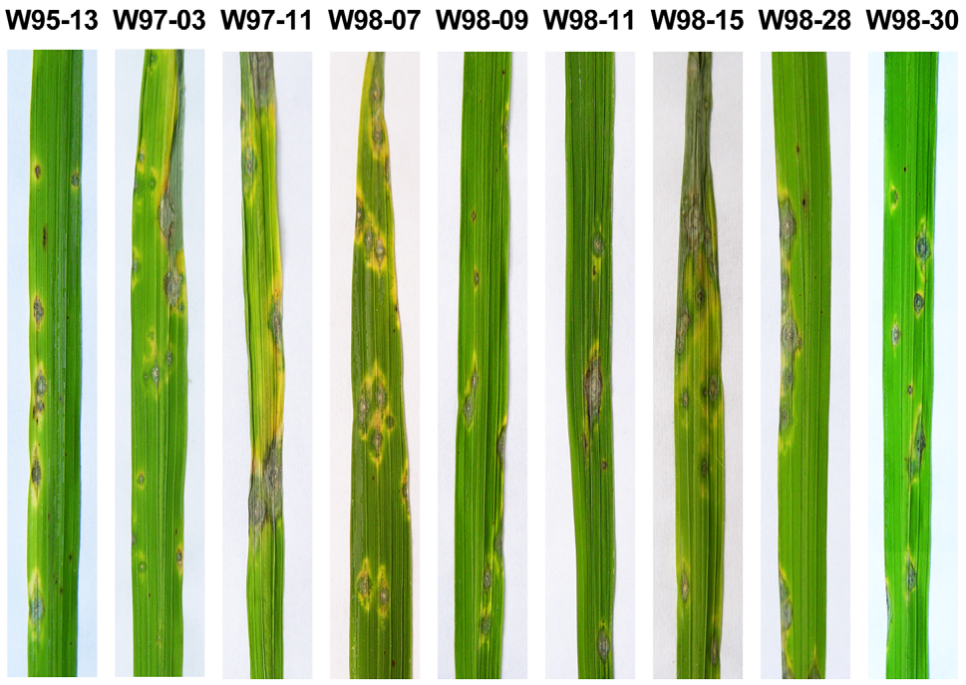
Pathogenicity of crabgrass isolates on rice. Pathogenicity assays were performed by spray inoculation (1×10^5^ spores/ml). Typical blast lesions were observed on the leaves 10 days after inoculation.

Large scale pathogenicity tests for *M. grisea* and *M. oryzae* isolates were performed to confirm cross-infectivity between rice and crabgrass. Leaves of rice and crabgrass were inoculated with 89 out of 103 isolates. The 89 isolates tested were divided into two groups for analysis: the first group consisted of 70 isolates from crabgrass and the second consisted of 19 from rice and other grasses. The first group from crabgrass showed diverse patterns in the virulence spectrum (Fig. 4 and Table 3). More specifically, 42 out of 70 crabgrass isolates (N_‘G’ type_: 40, N_‘O2’ type_: 2) produced typical lesions on rice leaves: 35 isolates (e.g. W97-11 and W98-27) showed consistent virulence (‘++’ or ‘+++’ in ‘Disease Index’ described in Materials and Methods) and seven (e.g. W95-03) successfully infected rice once out of three trials. Interestingly, two isolates with the ‘O2’ haplotype were consistently virulent on rice. The other 28 isolates (e.g. W97-06) failed to produce blast lesion on rice in any of the tests. As expected, all crabgrass isolates could infect crabgrass with ‘++’ or ‘+++’ index types. Thus, in the first group, 42 crabgrass isolates were pathogenic to both crabgrass and rice while the other 28 crabgrass isolates were pathogenic only to crabgrass. In the second group from rice and other grasses, three rice isolates could infect both rice and crabgrass with ‘+++’ index type. Sixteen isolates from other grasses (N_‘O’ type_: 8, N_‘N’ type_: 5, N_‘G’ type_: 3) were virulent with ‘++’ or ‘+++’ types for crabgrass. However, only five out of these 16 isolates (N_‘O’ type_:1, N_‘N’ type_:2, N_‘G’ type_:2) exhibited strong virulence on rice such as ‘++’ or ‘+++’ index types (Tables 3 and S1).

**Table 3:** Pathogenicity of *Mg* complex isolates on rice and crabgrass.

Groups of isolates	Disease index^a^	No. of isolates pathogenic on	
		Rice (%)	Crabgrass (%)
Crabgrass isolates (N = 70)	+++	18 (26%)	69 (99%)
	++	17 (24%)	1 (1%)
	+	7 (10%)	0 (0%)
	−	28 (40%)	0 (0%)
Rice and other grasses isolates (N = 19)	+++	5 (26%)	15 (79%)
	++	3 (16%)	4 (21%)
	+	7 (37%)	0 (0%)
	−	4 (21%)	0 (0%)

a Isolates exhibiting susceptible lesions (type 2 to 5) [44] were marked as ‘+’ and the other types (0 and 1) as resistant (‘−’). Disease incidence was displayed with numbers of ‘+’ marks in three trials (see MATERIALS AND METHODS).

**Figure 5:**
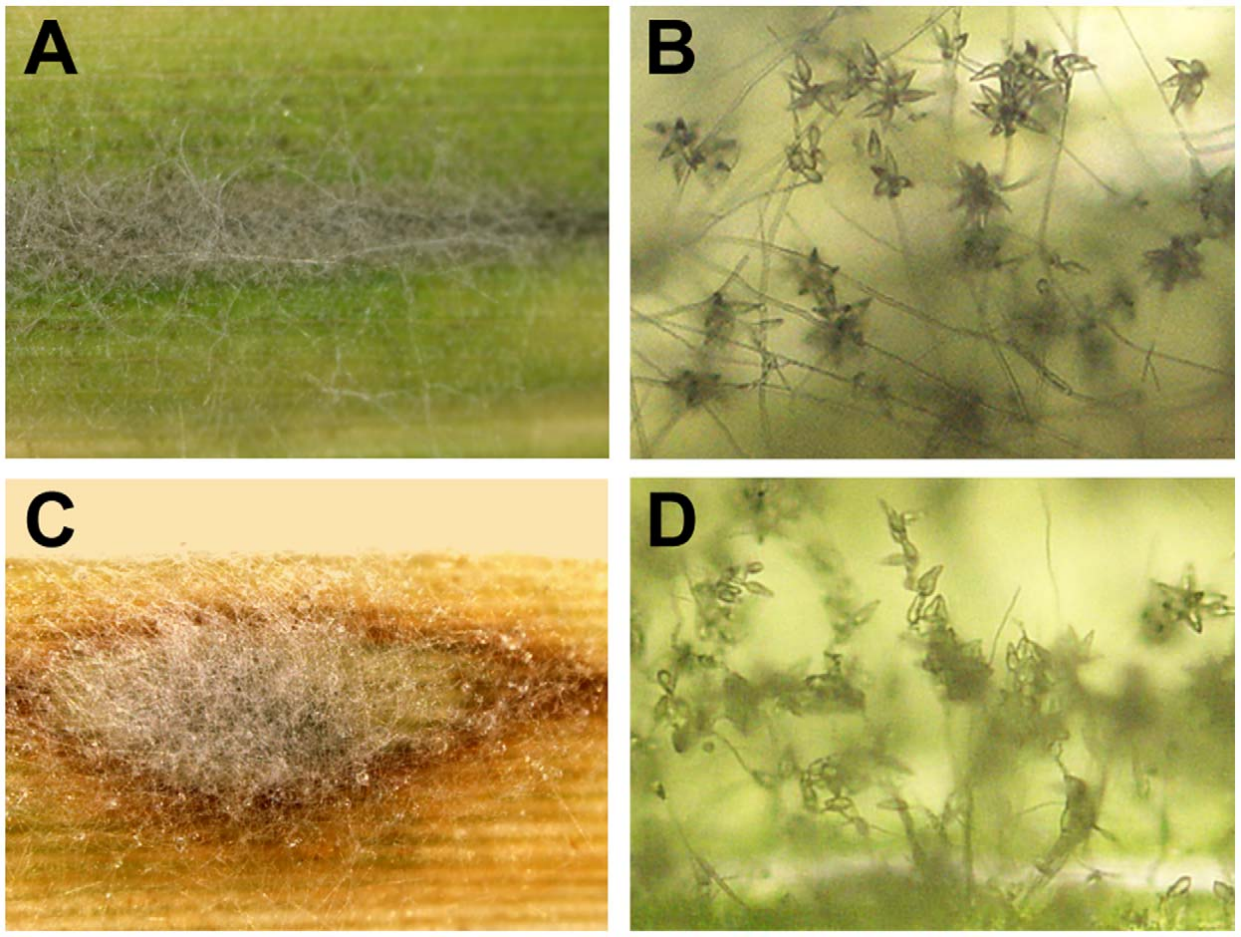
Conidial production on blast lesions. A–B. Conidia and conidiophore produced on lesion of rice leaf infected with conidia of the crabgrass isolates (W97-14). **C–D.** Lesion on crabgrass leaf showing conidia of the isolate from ryegrass (W95-11).

To further verify the cross-infectivity between rice and crabgrass, conidial production was observed on the lesions of both rice and crabgrass. The infected leaves were stored on water agar media under high humidity conditions as shown in Fig. 5. Gray mycelia grew over the lesions on the leaves and conidia were produced at the tips of conidiophores (Fig. 5). From this observation, we concluded that cross-infectivity exists between crabgrass and rice by *M. oryzae* and *M. grisea* isolates.

## Discussion

We performed phylogenetic analysis and pathogenicity assays for the blast pathogens isolated from rice, crabgrass, and other grasses surrounding rice fields to understand the structure of the *Mg* complex and its pathogenicity to rice and crabgrass. Together with phylogenetic analysis, cross-infection between crabgrass and rice in the field environment seemed plausible given the discovery of six isolates that exhibited inconsistency in haplotype-host origin relationship. This was confirmed by large-scale pathogenicity tests showing cross-infectivity of these isolates to crabgrass and rice.

In previous studies, only one or two crabgrass isolates were included in cross-inoculation assays, due to emphasis on rice pathogens [2], [23], [37], [38], [43], [53]. Moreover, the same strain (Dig4-1) was used repeatedly in different analyses [23], [38], [53]. Our aim was to comprehensively elucidate the *Mg* complex diversity in the field environment. An unprecedented collection of 103 isolates was obtained from different locations (N = 49) and years (N = 4) to provide a more representative picture of the actual pathogen diversity (Table S1). This collection allowed us to overcome the possible limitation and bias by small sample numbers shown in previous studies.

All isolates were identified genetically with the same multilocus genealogy method used to resolve *M. grisea* and *M. oryzae* groups [30]. Haplotypes of most isolates were associated with the host origin as expected. Sixty eight out of 70 crabgrass isolates (97.1%) were identified as having the *M. grisea* haplotype. Twenty-four out of 28 isolates from rice and other grasses (85.7%) possessed the *M. oryzae* haplotype. Thus, our results largely support the division of *M. grisea* and *M. oryzae* base on PSC [30]. However, there were three findings that extend our current understanding of genetic diversity within this complex.

First, a new lineage was found in the phylogenetic analysis and named the *Neo* group (Fig. 1). This group consists of five isolates from five different hosts (Table S1). The *Neo* group shares the same sequence with the ‘L&S’ group which was isolated from *Leersia oryzoides* and *Setaria geniculata* in a previous study [32]. The *Neo* and L&S groups can be combined and regarded as another phylogenetic species that consists of the seven isolates from seven different hosts of other grasses. However, considerable difficulty exists in identifying clear boundaries to delimit hosts of the *M. oryzae* and *Neo* groups. Although the phylogenetic positions in MPTs inferred from three individual genes were not congruent (Fig. S1), they have similar evolutionary distances to each other, suggesting that all three are independent phylogenetic species (Table 2).

Secondly, six isolates showed inconsistency in the haplotype-host origin relationship (Fig. 3 and Table 1). Two crabgrass isolates, W95-06 and W06-20, were discovered as having the *M. oryzae* haplotype (specifically ‘O2’ type) while one rice isolate (YHL-684) and three isolates from other grasses (W95-12, W97-16, and W97-17) had the *M. grisea* haplotype. As reported [30],the crabgrass isolate (94-118-1a) from China had the *M. oryzae* haplotype. Couch *et al*. [54] also found exceptional cases where two barley isolates had rice haplotypes (the haplotype H10; N = 123) and one rice isolate (H26) belonging to the clade formed by all goosegrass isolates (H24-5, H27-8, H30, H36-7; N = 19). The origins of these isolates should be reexamined by alternative methods such as a repetitive sequence hybridization analysis. Interestingly, this inconsistency of haplotype and host origin was observed only in the Asian isolates from Korea, China, and India [30], [54]. The reason for this may be the long history of rice cultivation over large areas during which time the *Mg* complex may have been presented with challenges that favored host shift or adaptation to various hosts.

Finally, 68 crabgrass isolates having the *M. grisea* haplotype also exhibited a broad range of virulence spectrum on rice (Tables 3 and S1), which clearly indicates cross-infectivity between crabgrass and rice. The fact that 28 out of 68 could not cause disease and the other 40 were virulent on rice might explain the contradictory results in the pathogenicity assays in previous studies where limited number of crabgrass isolates (one or two) were tested [2], [23], [37], [38], [42], [43]. On the other hand, most isolates from other grasses (N_‘O’ type_: 8, N_‘N’ type_: 5) were weakly virulent or avirulent on rice (No disease: 3, +: 7, ++: 1, +++:2). Similarly, other grasses isolates (except rice) caused less or no virulence toward rice in the previous pathogenicity test [54]. From these observations, the crabgrass isolates seemed to be more virulent to rice than the isolates from other grasses, suggesting a closer relationship of crabgrass isolates to rice isolates than isolates from other grasses in pathogenicity. Given cross-infectivity on rice and crabgrass, we hypothesize that crabgrass could be an overwintering shelter for *M. oryzae* in rice fields and *M. grisea* may be the primary inoculum of rice blast.

Recent applications of PSC are based on Genealogical Concordance Phylogenetic Species Recognition (GCPSR) that recognizes species as “independent and genealogically exclusive lineages that are typically resolved by phylogenetic analysis of multiple gene genealogies” [10], [55]. PSC has been applied extensively to species complexes within the Fungi that were difficult to resolve with MSC and BSC because PSC gives better resolutions [10], [29]. Such is the case for the *Mg* complex which cannot be subdivided easily by morphological or biological means. The phylogenetic analysis resolved that *M. oryzae* and *M. grisea* are not divergent lineages within the same species, but are independent species [30]. Subsequently, Zellerhoff *et al*. (2006) added a putative species in association with *Pennisetum* spp. closely related to *M. grisea* [31]. Five more groups including *P. zingiberi* and *P. zizaniaecola* were proposed in addition to this species [32]. A total of 37 haplotypes were identified in the *M. oryzae* species complex alone when ten marker sequences were used [54]. Application of PSC with more markers and samples will allow for a higher resolution of the *Mg* complex. However, all of these haplotypes cannot be regarded as individual species. While a few phylogenetic species in the *Mg* complex are also supported by MSC, as mentioned in the work of Hirata and colleagues [32], most phylogenetic species are morphologically indistinguishable. More conclusive methods are required to standardize the process of determining species limits within the *Mg* complex. Furthermore, it is necessary to select standard strains representing divergent haplotypes and independent and polymorphic loci, because the strains and loci used by many researchers are not standardized [15], [30], [32], [54], [56]. Towards this end, it may prove to be helpful to resequence genomes of the related species such as the *M. grisea* and *Neo* groups and to compare with the fully sequenced *M. oryzae*, *M. salvinii* and *M. poae* genomes.

Taken together, cross-infection or host shift in the *Mg* complex between crabgrass and rice was suggested from the inconsistent relationship between haplotypes and host origins of the isolates, and was further supported by comparative pathogenicity tests using both rice and crabgrass.

## Supporting Information

Figure S1 The maximum parsimony trees of *Mg* complex isolates inferred from actin, beta-tubulin, and calmodulin genes. Labels on the phylogeny are, from left to right: Strain no., host, and the phylogenetic group or species. Sequences used in a previous study [30] were integrated as controls (gray characters). Samples showing inconsistency in haplotype-host origin are indicated in red. (A) The single most parsimonious tree (MPT) was inferred from the actin gene. The tree length was 323 steps and the consistency index (CI) was 0.978. (B) The single MPT was inferred from the beta-tubulin gene. The tree length was 142 and the CI was 0.924. (C) The single MPT was inferred from the calmodulin gene. The tree length was 343 and the CI was 0.955. Bootstrap values, based on 500 replicates, are indicated above the branches.
(PPT)

Table S1 The isolates used in this study.
(DOC)
