# Supplementary material for: Comparative Analysis of Pathogenicity and Phylogenetic Relationship in Magnaporthe grisea Species Complex
Source: PLoS One. 2013 Feb 26;8(2):e57196. doi: 10.1371/journal.pone.0057196 (PMC3582606; doi:10.1371/journal.pone.0057196)
Supplement: Figure S1 — The maximum parsimony trees of Mg complex isolates inferred from actin, beta-tubulin, and calmodulin genes. Labels on the phylogeny are, from left to right: Strain no., host, and the phylogenetic group or species. Sequences used in a previous study [30] were integrated as controls (gray characters). Samples showing inconsistency in haplotype-host origin are indicated in red. (A) The single most parsimonious tree (MPT) was inferred from the actin gene. The tree length was 323 steps and the consistency index (CI) was 0.978. (B) The single MPT was inferred from the beta-tubulin gene. The tree length was 142 and the CI was 0.924. (C) The single MPT was inferred from the calmodulin gene. The tree length was 343 and the CI was 0.955. Bootstrap values, based on 500 replicates, are indicated above the branches. (PPT) [file pone.0057196.s001.ppt]

## Slide 1
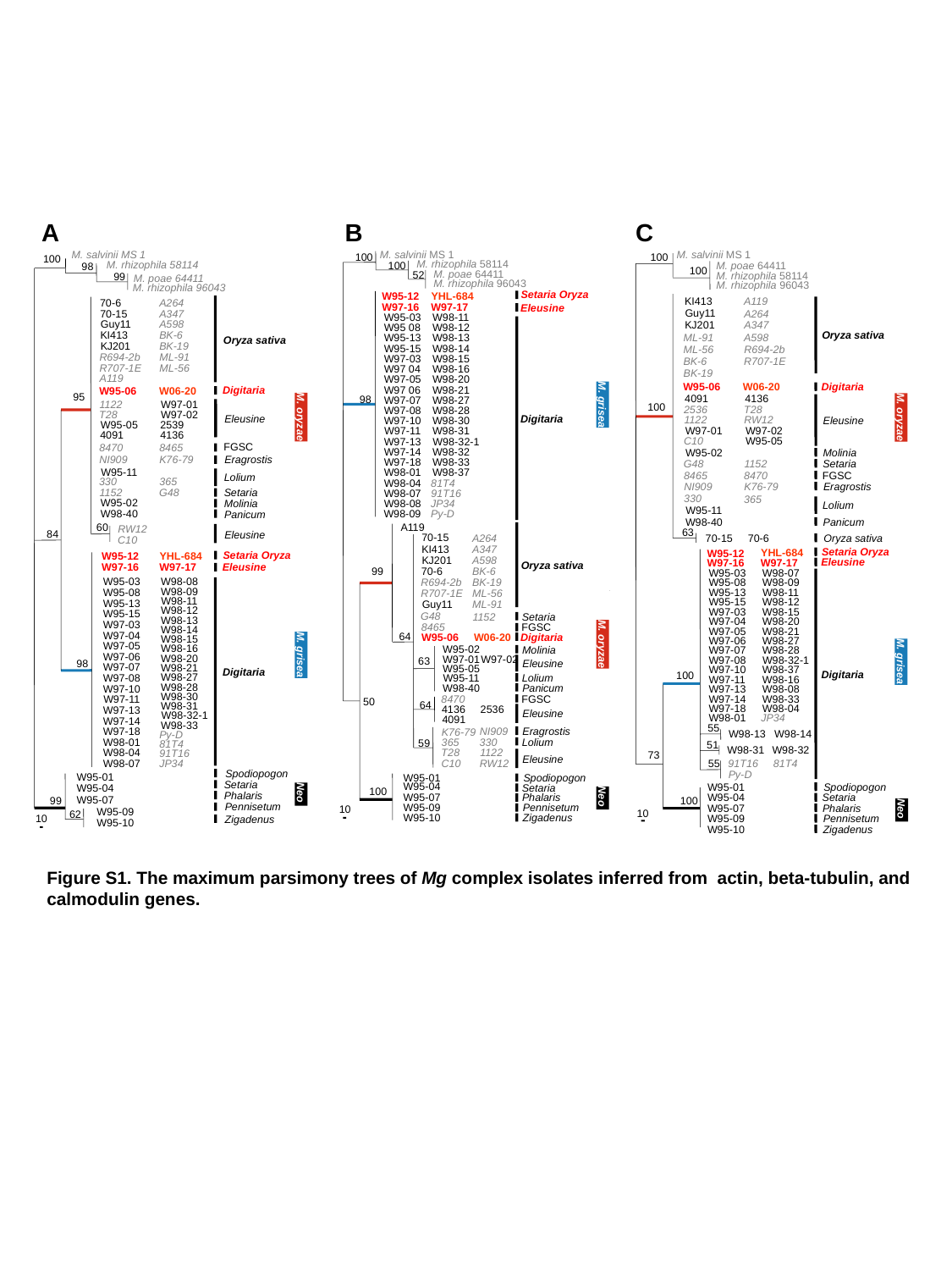

A
B
C
M. salvinii MS 1
100
M. rhizophila 58114
98
99
M. poae 64411
M. rhizophila 96043
70-6
A264
70-15
A347
A598
Guy11
BK-6
KI413
Oryza sativa
KJ201
BK-19
R694-2b
ML-91
R707-1E
ML-56
A119
Digitaria
W95-06
95
1122
W97-01
T28
W97-02
M. oryzae
Eleusine
W95-05
2539
4091
4136
FGSC
8470
8465
NI909
K76-79
Eragrostis
W95-11
Lolium
330
365
1152
G48
Setaria
W95-02
Molinia
W98-40
Panicum
60
RW12
84
Eleusine
C10
Setaria Oryza
W95-12
W97-16
W97-17
Eleusine
W95-03
W98-08
W98-09
W95-08
W98-11
W95-13
W98-12
W95-15
W98-13
W97-03
W98-14
W97-04
W98-15
W97-05
W98-16
M. grisea
W97-06
W98-20
98
W97-07
W98-21
Digitaria
W98-27
W97-08
W98-28
W97-10
W98-30
W97-11
W98-31
W97-13
W98-32-1
W97-14
W98-33
W97-18
Py-D
W98-01
81T4
W98-04
91T16
W98-07
JP34
Spodiopogon
W95-01
W95-04
W95-07
W95-09
W95-10
Setaria
Neo
Phalaris
99
Pennisetum
62
10
Zigadenus
M. salvinii MS 1
100
M. rhizophila 58114
100
M. poae 64411
52
M. rhizophila 96043
Setaria Oryza
W95-12
W97-16
W97-17
Eleusine
W95-03
W98-11
W95 08
W98-12
W95-13
W98-13
W95-15
W98-14
W97-03
W98-15
W97 04
W98-16
W97-05
W98-20
W97 06
W98-21
98
W97-07
W98-27
W97-08
W98-28
Digitaria
W97-10
W98-30
W97-11
W98-31
W97-13
W98-32-1
W97-14
W98-32
W97-18
W98-33
W98-01
W98-37
W98-04
81T4
W98-07
91T16
W98-08
JP34
Py-D
W98-09
A119
70-15
A264
KI413
A347
KJ201
A598
Oryza sativa
70-6
BK-6
99
R694-2b
BK-19
R707-1E
ML-56
Guy11
ML-91
G48
1152
Setaria
FGSC
8465
64
W95-06
Digitaria
W95-02
Molinia
W97-01
W97-02
63
Eleusine
W95-05
W95-11
Lolium
Panicum
W98-40
FGSC
8470
50
64
4136
2536
Eleusine
4091
Eragrostis
NI909
K76-79
365
330
Lolium
59
T28
1122
Eleusine
C10
RW12
W95-01
Spodiopogon
W95-04
Setaria
100
W95-07
Phalaris
W95-09
Pennisetum
10
W95-10
Zigadenus
M. grisea
M. oryzae
Neo
M. salvinii MS 1
100
M. poae 64411
100
M. rhizophila 58114
M. rhizophila 96043
KI413
A119
Guy11
A264
KJ201
A347
Oryza sativa
ML-91
A598
ML-56
R694-2b
BK-6
R707-1E
BK-19
W95-06
Digitaria
4091
4136
100
2536
T28
1122
RW12
Eleusine
W97-01
W97-02
C10
W95-05
W95-02
Molinia
Setaria
G48
1152
8465
8470
FGSC
NI909
K76-79
Eragrostis
330
365
Lolium
W95-11
W98-40
Panicum
63
70-15
70-6
Oryza sativa
Setaria Oryza
W95-12
Eleusine
W97-16
W97-17
W95-03
W98-07
W95-08
W98-09
W95-13
W98-11
W95-15
W98-12
W97-03
W98-15
W97-04
W98-20
W97-05
W98-21
W97-06
W98-27
W97-07
W98-28
W97-08
W98-32-1
W97-10
W98-37
Digitaria
100
W97-11
W98-16
W97-13
W98-08
W97-14
W98-33
W97-18
W98-04
W98-01
JP34
55
W98-13
W98-14
51
W98-31
W98-32
73
91T16
81T4
55
Py-D
W95-01
Spodiopogon
W95-04
Setaria
100
W95-07
Phalaris
10
W95-09
Pennisetum
W95-10
Zigadenus
M. oryzae
M. grisea
Neo
YHL-684
W06-20
W06-20
YHL-684
YHL-684
W06-20
Figure S1. The maximum parsimony trees of Mg complex isolates inferred from actin, beta-tubulin, and calmodulin genes.
