# Supplementary material for: Comparative Analysis of Pathogenicity and Phylogenetic Relationship in Magnaporthe grisea Species Complex
Source: PLoS One. 2013 Feb 26;8(2):e57196. doi: 10.1371/journal.pone.0057196 (PMC3582606; doi:10.1371/journal.pone.0057196)
Supplement: Table S1 — The isolates used in this study. (DOC) [file pone.0057196.s002.doc]

**Table S1. The isolates** used in this study

| Strain No. | Isolated Hosts | Locations | Haplotypes^a^ | Disease Index ^b^ | |
| --- | --- | --- | --- | --- | --- |
|  |  |  |  | Rice | Crabgrass |
| W06-03 | *Digitaria sanguinalis* | Gyeonggi | G | +++^c^ | +++ |
| W06-29 | *Digitaria sanguinalis* | Gyeonggi | G | +++^c^ | +++ |
| W06-37 | *Digitaria sanguinalis* | Chungbuk | G | +++^c^ | +++ |
| W95-08 | *Digitaria sanguinalis* | Gyeongnam | G | +++ | +++ |
| W97-04 | *Digitaria sanguinalis* | Gyeonggi | G | +++ | +++ |
| W97-11 | *Digitaria sanguinalis* | Chungbuk | G | +++ | +++ |
| W97-13 | *Digitaria sanguinalis* | Gyeongbuk | G | +++ | +++ |
| W98-04 | *Digitaria sanguinalis* | Jeonnam | G | +++ | +++ |
| W98-07 | *Digitaria sanguinalis* | Gyeongnam | G | +++ | +++ |
| W98-08 | *Digitaria sanguinalis* | Gyeongnam | G | +++ | +++ |
| W98-12 | *Digitaria sanguinalis* | Gyeonggi | G | +++ | +++ |
| W98-27 | *Digitaria sanguinalis* | Chungbuk | G | +++ | +++ |
| W98-28 | *Digitaria sanguinalis* | Chungbuk | G | +++ | +++ |
| W98-30 | *Digitaria sanguinalis* | Gyeonggi | G | +++ | +++ |
| W98-32-1 | *Digitaria sanguinalis* | Gyeonggi | G | +++ | ++ |
| W95-13 | *Digitaria sanguinalis* | Gyeonggi | G | ++ | +++ |
| W97-03 | *Digitaria sanguinalis* | Gyeonggi | G | ++ | +++ |
| W97-05 | *Digitaria sanguinalis* | Gyeongnam | G | ++ | +++ |
| W97-07 | *Digitaria sanguinalis* | Chungbuk | G | ++ | +++ |
| W97-08 | *Digitaria sanguinalis* | Chungbuk | G | ++ | +++ |
| W97-16 | *Eleusine indica* | Gyeongbuk | G | ++ | +++ |
| W97-17 | *Eleusine indica* | Gyeongbuk | G | ++ | +++ |
| W97-18 | *Digitaria sanguinalis* | Gyeongnam | G | ++ | +++ |
| W98-01 | *Digitaria sanguinalis* | Gyeongnam | G | ++ | +++ |
| W98-09 | *Digitaria sanguinalis* | Gyeongbuk | G | ++ | +++ |
| W98-11 | *Digitaria sanguinalis* | Gyeonggi | G | ++ | +++ |
| W98-13 | *Digitaria sanguinalis* | Gyeonggi | G | ++ | +++ |
| W98-14 | *Digitaria sanguinalis* | Chungbuk | G | ++ | +++ |
| W98-15 | *Digitaria sanguinalis* | Chungbuk | G | ++ | +++ |
| W98-16 | *Digitaria sanguinalis* | Gangwon | G | ++ | +++ |
| W98-20 | *Digitaria sanguinalis* | Chungnam | G | ++ | +++ |
| W98-32 | *Digitaria sanguinalis* | Gyeonggi | G | ++ | +++ |
| W98-33 | *Digitaria sanguinalis* | Chungbuk | G | ++ | +++ |
| W95-03 | *Digitaria sanguinalis* | Chungnam | G | + | +++ |
| W95-15 | *Digitaria sanguinalis* | Chungnam | G | + | +++ |
| W97-10 | *Digitaria sanguinalis* | Chungbuk | G | + | +++ |
| W97-14 | *Digitaria sanguinalis* | Gyeongbuk | G | + | +++ |
| W98-21 | *Digitaria sanguinalis* | Gyeongnam | G | + | +++ |
| W98-31 | *Digitaria sanguinalis* | Gyeonggi | G | + | +++ |
| W98-37 | *Digitaria sanguinalis* | Gyeonggi | G | + | +++ |
| W97-06 | *Digitaria sanguinalis* | Chungbuk | G | - | +++ |
| W06-01 | *Digitaria sanguinalis* | Gyeonggi | G | -^c^ | +++ |
| W06-02 | *Digitaria sanguinalis* | Gyeonggi | G | -^c^ | +++ |
| W06-04 | *Digitaria sanguinalis* | Gyeonggi | G | -^c^ | +++ |
| W06-05 | *Digitaria sanguinalis* | Gyeonggi | G | -^c^ | +++ |
| W06-06 | *Digitaria sanguinalis* | Gyeonggi | G | -^c^ | +++ |
| W06-07 | *Digitaria sanguinalis* | Gyeonggi | G | -^c^ | +++ |
| W06-08 | *Digitaria sanguinalis* | Gyeonggi | G | -^c^ | +++ |
| W06-09 | *Digitaria sanguinalis* | Gyeonggi | G | -^c^ | +++ |
| W06-10 | *Digitaria sanguinalis* | Gyeonggi | G | -^c^ | +++ |
| W06-11 | *Digitaria sanguinalis* | Gyeonggi | G | -^c^ | +++ |
| W06-12 | *Digitaria sanguinalis* | Gyeonggi | G | -^c^ | +++ |
| W06-13 | *Digitaria sanguinalis* | Gyeonggi | G | -^c^ | +++ |
| W06-14 | *Digitaria sanguinalis* | Gyeonggi | G | -^c^ | +++ |
| W06-19 | *Digitaria sanguinalis* | Chungbuk | G | -^c^ | +++ |
| W06-21 | *Digitaria sanguinalis* | Gyeonggi | G | -^c^ | +++ |
| W06-22 | *Digitaria sanguinalis* | Gyeonggi | G | -^c^ | +++ |
| W06-23 | *Digitaria sanguinalis* | Gyeonggi | G | -^c^ | +++ |
| W06-24 | *Digitaria sanguinalis* | Gyeonggi | G | -^c^ | +++ |
| W06-25 | *Digitaria sanguinalis* | Gyeonggi | G | -^c^ | +++ |
| W06-26 | *Digitaria sanguinalis* | Gyeonggi | G | -^c^ | +++ |
| W06-27 | *Digitaria sanguinalis* | Gyeonggi | G | -^c^ | +++ |
| W06-28 | *Digitaria sanguinalis* | Gyeonggi | G | -^c^ | +++ |
| W06-30 | *Digitaria sanguinalis* | Gyeonggi | G | -^c^ | +++ |
| W06-31 | *Digitaria sanguinalis* | Gyeonggi | G | -^c^ | +++ |
| W06-32 | *Digitaria sanguinalis* | Chungbuk | G | -^c^ | +++ |
| W06-33 | *Digitaria sanguinalis* | Chungbuk | G | -^c^ | +++ |
| W06-34 | *Digitaria sanguinalis* | Chungbuk | G | -^c^ | +++ |
| W06-35 | *Digitaria sanguinalis* | Chungbuk | G | -^c^ | +++ |
| W06-36 | *Digitaria sanguinalis* | Chungbuk | G | -^c^ | +++ |
| W95-12 | *Setaria viridis* | Gyeongbuk | G | - | ++ |
| YHL-684 | *Oryzae sativa* | Jeonnam | G | NA | NA |
| W95-07 | *Phalaris arundinacea* | Gyeonggi | N | +++ | +++ |
| W95-04 | *Setaria viridis* | Gyeonggi | N | ++ | +++ |
| W95-01 | *Spodiopogon sibiricus* | Gyeonggi | N | + | +++ |
| W95-10 | *Zigadenus elegans* | Gyeonggi | N | + | +++ |
| W95-09 | *Pennisetum clandestinum* | Gyeonggi | N | - | ++ |
| 4136-4-3 | *Eleusine* X *Eragrostis* | Lab strain | O | +++ | +++ |
| W97-01 | *Eleusine indica* | Gyeonggi | O | + | +++ |
| W97-02 | *Eleusine indica* | Gyeonggi | O | + | +++ |
| W98-40 | *Panicum miliaceum* | Chungbuk | O | + | +++ |
| 4091-5-8 | *Eleusine* X *Eragrostis* | Lab strain | O | - | +++ |
| W95-11 | *Lolium sp.* | Gyeonggi | O | - | +++ |
| W95-02 | *Molinia japonica* | Gyeonggi | O | + | ++ |
| W95-05 | *Eleusine indica* | Jeonnam | O | + | ++ |
| 2539 | *Oryza* X *Eleusine* | Lab strain | O | NA | NA |
| 70-15 | *Oryza* X *Eleusine* | Lab strain | O2 | +++ | +++ |
| Guy11 | *Oryzae sativa* | Guyana | O2 | +++ | +++ |
| KI413 | *Oryzae sativa* | Jeonbuk | O2 | +++ | +++ |
| W06-20 | *Digitaria sanguinalis* | Gyeonggi | O2 | +++^c^ | +++ |
| W95-06 | *Digitaria sanguinalis* | Gyeongnam | O2 | ++ | +++ |
| 70-6 | *Oryza* X *Eleusine* | Lab strain | O2 | NA | NA |
| KJ201 | *Oryzae sativa* | Jeonbuk | O2 | NA | NA |
| YHL-724 | *Oryzae sativa* | Gangwon | O2 | NA | NA |
| YHL-747 | *Oryzae sativa* | Chungbuk | O2 | NA | NA |
| YHL-754 | *Oryzae sativa* | Chungnam | O2 | NA | NA |
| YHL-775 | *Oryzae sativa* | Gyeonggi | O2 | NA | NA |
| YHL-873 | *Oryzae sativa* | Jeonbuk | O2 | NA | NA |
| YHL-897 | *Oryzae sativa* | Jeonbuk | O2 | NA | NA |
| YHL-908 | *Oryzae sativa* | Gangwon | O2 | NA | NA |
| YHL-910 | *Oryzae sativa* | Gyeonggi | O2 | NA | NA |
| YHL-911 | *Oryzae sativa* | Chungbuk | O2 | NA | NA |
| YHL-912 | *Oryzae sativa* | Gyeongnam | O2 | NA | NA |

^a^ G: *M. grisea*, N: *Neo* group, O: *M. oryzae,* O2: *M. oryzae* from rice (4 nt missing compared to O type)

^b^ Isolates exhibiting susceptible lesions (type 2 to 5) [44] were marked as ‘+’ and the other types (0 and 1) as resistant (‘-’). Disease incidence was displayed with numbers of ‘+’ marks in three trials (see MATERIALS AND METHODS).

^c^ One pathogenicity test was done to the susceptible cultivar Nakdongbyeo.
